# Safety and benefits of large-volume liposuction: a single center experience

**DOI:** 10.1186/1755-7682-2-4

**Published:** 2009-02-02

**Authors:** Youssef Saleh, Mahmoud El-Oteify, Abd-El-Radi Abd-El-Salam, Ahmed Tohamy, Alaa A Abd-Elsayed

**Affiliations:** 1 Plastic Surgery Department, Assiut University Hospital, Assiut University, Assiut, Egypt; 2 General Surgery Department, Assiut University Hospital, Assiut University, Assiut, Egypt; 3 Public Health and Community Medicine Department, Faculty of Medicine, Assuit University, Assiut, Egypt

## Abstract

**Background:**

Liposuction is a surgical technique to remove excess fat deposits from specific areas of the body. Purpose of this study is to determine how far large volume liposuction is safe and effective.

**Methodology:**

From July 2003 to December 2005, 60 female patients had liposuction of different areas of the body as waist, hips, buttocks, thighs, and knees. Their mean age was 30.6 ± 15.4 years old. A standard liposuction technique was done by using a tumescent infiltration formula. The average amount of infusate was 3000 cc, with an average aspirate amount of 6000 cc. Pre-operative anthropometric measurements as weight, height, body mass index, areas to be liposuctioned in addition to pre-operative hematological investigations as complete blood picture, blood sugar, liver function tests, blood urea, serum creatinine, and serum cholesterol were done.

**Results:**

The results were evaluated with preoperative and postoperative photographs. Postoperative anthropometric measurements and hematological investigations were done at 6^th ^week, and 4^th ^month after surgery. The rate of complications was low and relatively minor in nature. No major complications were presented. Minor complications have occurred as skin irregularities (20%), Seroma (15%), Garment pressure sore (10%), Cutaneous hyper-pigmentation (5%).

**Conclusion:**

Large-volume liposuction can be performed safely and it can produce desirable morphological and hematological changes.

## Introduction

Obesity is a growing health problem. Excess weight and obesity increase morbidity and mortality associated with numerous complications, including type 2 diabetes mellitus, hyperlipidemia, hypertension and atherosclerosis [1]. Liposuction is the most common surgical procedure for obesity in the world. Relative ease of performance, high patient satisfaction rate, and relatively high safety of the procedure are reasons for its popularity.

It removes excess fat deposits from specific areas of the body, including chin, neck, cheeks, and upper arms, above the breasts, abdomen, buttocks, hips, thighs, knees, calves and ankles [2]. The best candidates for liposuction are of relatively normal weight with firm elastic skin, but have pockets of excess fat in certain areas. The candidate should be physically healthy, psychologically stable and realistic in expectations [3].

There are different techniques of liposuction such as dry, wet, super wet and tumescent techniques. Tumescent technique is considered a significant improvement in liposuction surgery. It uses large volumes of a dilute solution of lidocaine, a local anesthetic, in combination with epinephrine. This technique dramatically reduces both the bleeding during surgery and the post-operative bruising and swelling [4].

The most common definitions of large volume liposuction refer to either total fat removal during the procedure (e.g. 4 L of fat removal) or total volume removed during the procedure (fat plus wetting solution, e.g. 5 L of total volume removal) [5].

As with any surgical procedure, liposuction is associated with certain expected side effects such as bruising, swelling and temporary numbness. Although irregularities of the skin are possible following liposuction, this is minimized by the tumescent technique [6]. Major complications tend to be rare and can be minimized by adhering to the 5 pillars of safety i.e. properly trained and educated surgeon in liposuction techniques, well trained anesthesiologist, completely equipped facility where the procedure is performed, trained support staff working in the operating room and recovery room, proper selection of the patient [6].

As judged by current worldwide experience, liposuction is amazingly safe. Serious complications as infections and allergic reactions are extremely rare.

## Methodology

• This study aimed at evaluating the degree of morphological improvement as well as hematological changes in patients undergoing large volume liposuction.

• Inclusion criteria, all females admitted to our plastic surgery department between July 1^st^, 2003 and December 31^st ^2005 with obesity as defined by the WHO (BMI > 30 kg/m^2^) [7], requesting liposuction, fit for the procedure and free from other co-morbidities as summarized in the exclusion criteria.

• They were presented by varying degrees of lipodystrophy in different body regions including (waist, hips, buttocks, thighs, and knees).

• Exclusion criteria, the presence of significant medical diseases such as diabetes (n = 7), cardiac (n = 5), renal (n = 4), hepatic (n = 2), gastrointestinal (n = 2), endocrinal diseases (n = 2) or body dysmorphic disorders (n = 3). These cases were excluded in order to avoid the potential confounding effects that these diseases might introduce.

• Each patient was subjected to full history, systematic examination.

• Pre-operative anthropometric measurements were taken for every patient including weight, height, body mass index, circumference of the areas to be lipsuctioned as waist, hips, buttocks, thighs, and knees.

• Pre-operative hematological investigations in the form of complete blood count, Blood sugar level, Blood urea and serum creatinine, Liver function tests (total protein, albumin, uric acid), and Serum cholesterol.

• Hemoglobin level and hematocrit value were measured using Celldyne automated cell counter, while blood glucose, urea, serum creatinine, total proteins, albumin, uric acid, total cholesterol were measured using Cobus Integra automated chemistry analyzer (Roche diagnostics kits). The same methods and kits were used for all patients and in all follow up measurements.

• Intra-operative local examination of the suctioned part was done by the use of the following tests: static pinching test, dynamic pinching test and the speed of retraction test.

### Operative details

• Our cases undergone Tumescent liposuction in which the surgeon injects a solution containing a local anesthetic and vasoconstrictor directly into the subcutaneous fat to be removed. The volume of fluid creates a space between the muscle and the fatty tissue allowing more room for the cannula.

• Spinal anesthesia was the rule in all cases.

• Intra-operative monitoring of the patients for oxygen and carbon dioxide tension, blood gases, electrocardiography, and urine output through insertion of urinary catheter.

• Tumescent formula (Modified Klein's solution) was used which consists of, 20 ml of 2% lidocaine, 1 ml adrenaline (1:1000), 5 ml of sodium bicarbonate solution (8.4%) and Mixed in 500 ml of lactated Ringer's solution.

• The injection was continued until the skin became firm and turge.

### Standard big five for better results

• Marking, positioning, and small stab incision.

• Pre-tunneling.

• Cross tunneling.

• Complete suction indicated when, pinch test less than 1 inch and/or change of feeling from soft to gritty sensation and/or aspirate mixed with blood.

• Pressure garment applied for 6 weeks [8].

### Post-operative Care

Broad spectrum antibiotics, non-steroidal anti-inflammatory analgesics twice daily for 2 days, multi-vitamins and iron supplements were routinely given to every patient for one month postoperatively, early ambulation from the second day after surgery, pressure garments for 6 weeks, post-operative massage and ultrasonic wave therapy starting at the 3^rd ^week, soothing corticosteroid creams for 6 weeks and strict diet regimen starting at the end of the 6^th ^week.

Repetition of the anthropometric and hematological measures at 6^th ^week and at the end of the 4^th ^month.

### Statistical analysis

SPSS version 13 was used for analysis of data which included descriptive analysis and paired t-test for comparison between measurements before the operation and 6 weeks after the operation and also comparison between measurements before the operation and 4 months after the operation.

## Results

This study included 60 females; their mean age was 30.6 ± 15.4 years old. Overall, patients' weight and body mass index decreased significantly both at 6-weeks post-operative period and at 4-months post-operative period (P < 0.001), table 1.

**Table 1:** Shows the mean values of body weight and body mass index over 4-months post-operative period and their significance:

	**Pre-operative**	**6 weeks post-operative**		**4 months post-operative**	
	**Mean ± S.D**.	**Mean ± S.D**.	*P *value*	**Mean ± S.D**.	*P *value**
**Weight (Kg)**	91.3 ± 17.6	82.6 ± 15.8	< 0.001^$#^	76.95 ± 14.9	< 0.001^$#^

**Body mass index**	34.95 ± 5.9	31.77 ± 5.6	< 0.001^$#^	29.69 ± 5.2	< 0.001^$#^

* Paired t-test for comparing preoperative with 6 weeks postoperative findings.

** Paired t-test for comparing preoperative with 4 months postoperative findings.

^# ^Significant difference at *p *value ≤ 0.05

^$ ^Significant difference at *p *value < 0.01

^$# ^Significant difference at *p *value < 0.001

**Figure 1:**
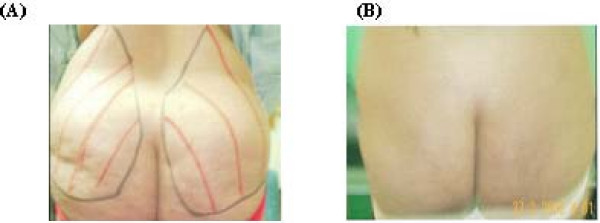
**A 42- year- old female patient with hip and buttocks lipodystrophy before surgery (A) and 4 months after surgery (B)**.

**Figure 2:**
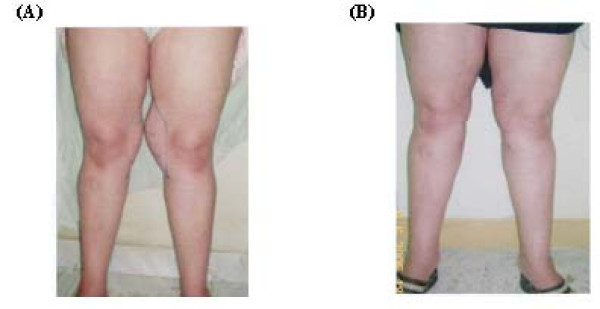
**A 36- year- old female patient with knee lipodystrophy before surgery (A) and 4 months after surgery (B)**.

**Figure 3:**
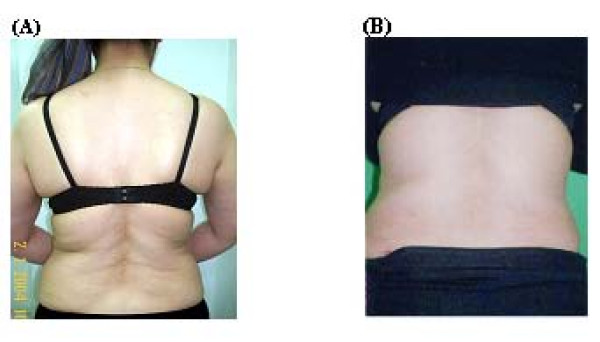
**A 31- year- old female patient with hip and back lipodystrophy before surgery (A) and 4 months after surgery (B)**.

**Figure 4:**
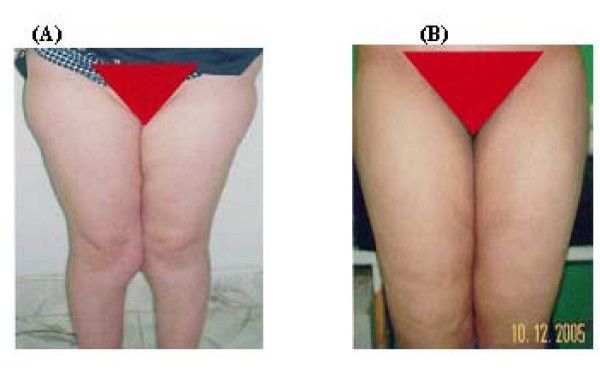
**A 46- year- old female patient with hip and thigh lipodystrophy before surgery (A) and 4 months after surgery (B)**.

**Figure 5:**
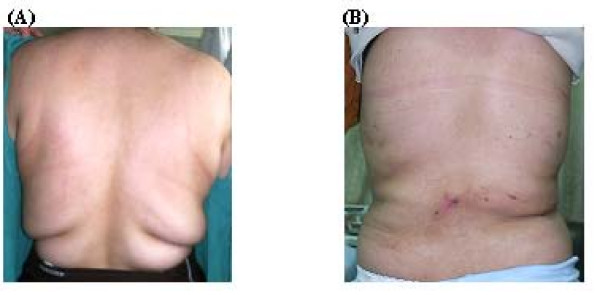
**A 39- year- old female patient with hip and back lipodystrophy before surgery (A) and 4 months after surgery (B)**.

The average amount of liposuction infusate was 2755 cc, with a mean aspirate amount of 6395 cc. Local examination and pre-operative evaluation of the areas of bulge of the patients was done to assess the areas in need for liposuction. We found that all the patients were in need for hip and thigh liposuction and fourteen patients were in need for waist liposuction, twelve patients for knee and four patients for buttocks liposuction in addition to hip and thigh suction, (figures 1, 2, 3, 4, 5). There is a significant decrease in different body measurements at both 6 weeks and 4 months after surgery, table 2.

**Table 2:** Shows the mean values of different body measurements over 4-months post-operative period and their significance:

**Body measurements (cm)**	**Pre-operative**	**6 weeks post-operative**		**4 months post- operative**	
	**Mean ± S.D**.	**Mean ± S.D**.	*P *value*	**Mean ± S.D**.	*P *value**
**Waist measurements**	102.6 ± 23.5	94.8 ± 20.1	0.001^$^	87 ± 17.2	0.005^$^
**Hip measurements**	115.8 ± 12.1	107.5 ± 10.9	< 0.001^$#^	101.9 ± 10.5	< 0.001^$#^
**Buttocks' measurements**	111 ± 11.5	106.8 ± 11.5	< 0.001^$#^	104.3 ± 11.1	0.001^$^
**Thigh measurements**	75.7 ± 10.7	69.4 ± 8.7	< 0.001^$#^	64.6 ± 8.1	< 0.001^$#^

**Knee measurements**	58.3 ± 6.4	52.5 ± 4.7	0.01^#^	48.3 ± 4.3	0.03^#^

* Paired t-test for comparing preoperative with 6 weeks postoperative findings.

** Paired t-test for comparing preoperative with 4 months postoperative findings.

^# ^Significant difference at *p *value ≤ 0.05

^$^Significant difference at *p *value < 0.01

^$#^Significant difference at *p *value < 0.001

The pulse rate was not decreased significantly at 6-weeks post-operatively but it showed a significant decrease at 4 months postoperatively. There was a significant decrease in systolic blood pressure both at 6 weeks and 4 months post-operatively while there was non-significant decrease in diastolic blood pressure both at 6-weeks and 4 months post-operatively, table 3.

**Table 3:** Shows the mean values of pulse rate (beats/minute) and blood pressure (mmHg) over 4-months post-operative period and their significance:

	**Pre-operative**	**6 weeks post-operative**		**4 months post- operative**	
	**Mean ± S.D**.	**Mean ± S.D**.	*P *value*	**Mean ± S.D**.	*P *value**
**Pulse rate**	83.15 ± 9.7	81.8 ± 8.9	NS	80.7 ± 9.2	0.02^#^
**Systolic B.P**.	128.3 ± 12.2	118.8 ± 10.9	< 0.001^$#^	116.5 ± 8.5	< 0.001^$#^

**Diastolic B.P**.	80.3 ± 8.5	79.3 ± 6.7	NS	79.5 ± 5.6	NS

* Paired t-test for comparing preoperative with 6 weeks postoperative findings.

** Paired t-test for comparing preoperative with 4 months postoperative findings.

^# ^Significant difference at *p *value ≤ 0.05

^$^Significant difference at *p *value < 0.01

^$#^Significant difference at *p *value < 0.001

Blood glucose level decreased non-significantly 6 weeks after surgery but it started to decrease significantly at 4 months post-operatively. Blood urea and serum creatinine levels decreased significantly both at 6 weeks and 4 months post-operatively.

There was a significant decrease in the total protein level, total cholesterol level and serum albumin level 6 weeks after surgery but gradually returned to near the pre-operative levels 4 months post-operatively. There was a significant decrease in the serum uric acid both at 6 weeks and 4 months post-operatively, table 4.

**Table 4:** Shows the mean values of laboratory investigations over 4-months post-operative period and their significance:

	**Pre-operative**	**6 weeks post-operative**		**4 months post-operative**	
	**Mean ± S.D**.	**Mean ± S.D**.	*P *value*	**Mean ± S.D**.	*P *value**
**Hemoglobin level** **(g/dl)**	12.1 ± 1.3	10.6 ± 1.3	< 0.001^$#^	12.1 ± 1.2	NS
**Hematocrit value**	36.5 ± 3	31.8 ± 4.1	< 0.001^$#^	36.7 ± 2.7	NS
**Blood glucose** **(mmol/L)**	5.3 ± 0.8	5.2 ± 1	NS	4.9 ± 0.6	0.005^$^
**Blood urea** **(mmol/L)**	4.7 ± 1.4	3.9 ± 1.7	< 0.001^$#^	4.5 ± 1.2	< 0.001^$#^
**Serum creatinine** **(μmol/L)**	70.6 ± 15.3	58.5 ± 11.9	< 0.001^$#^	64.6 ± 11.9	< 0.001^$#^
**Serum total protein** **(g/dl)**	75.5 ± 5.1	65.6 ± 7	< 0.001^$#^	74.4 ± 4.4	NS
**Serum albumin** **(g/dl)**	41.8 ± 3.6	35.9 ± 3	< 0.001^$#^	42.3 ± 3.9	NS
**Serum uric acid** **(mg/dl)**	5.1 ± 1.5	4.6 ± 1.4	< 0.001^$#^	4.3 ± 1.4	< 0.001^$#^

**Total cholesterol (mg/dl)**	170.5 ± 35.8	162.4 ± 32.5	< 0.001^$#^	167.4 ± 35.2	NS

* Paired t-test for comparing preoperative with 6 weeks postoperative findings.

** Paired t-test for comparing preoperative with 4 months postoperative findings.

^#^Significant difference at *p *value ≤ 0.05

^$^Significant difference at *p *value < 0.01

^$#^Significant difference at *p *value < 0.001

Cases did not present with any major complications but only some of them presented with minor complications as skin irregularities in 20% of the patients, seroma in 15%, garment pressure sore in 10% and cutaneous hyperpigmentation in 5%.

## Discussion

Since liposuction was initially introduced to the surgical community, its applications have progressively broadened from its use in small regional operations to total body contouring. The tumescent technique uses a large volume of vasoconstrictive subcutaneous infiltration and produces the greatest degree of hemostasis. Approximately 1% of the aspirate is blood [9]. In our study, the average amount of infusate was 3000 c.c., with an average aspirate amount of 6000 c.c. Our results, as well as those of several other investigators, have demonstrated the same findings [5,10,11].

No blood transfusion was needed postoperatively for any of our cases. Paul et al [12] who performed total body contouring with mega-liposuction of 8 liters and more on 120 consecutive cases without the need for blood transfusion.

Large-volume liposuction caused significant decreases in pulse rate and systolic blood pressure over the 4-month post-operative period. The same was found by many authors [13-15] who reported that there were significant decreases in pulse rate and systolic blood pressure over the 4-months post-operative period. We hypothesize that salutary physiologic and metabolic changes occur secondary to enhanced insulin sensitivity and weight loss. Circulating insulin has previously been suggested to be an important factor affecting blood pressure and to be a predictor of later cardiac dysfunction [16,17].

Our results showed significant decrease in the blood glucose level after 4 months postoperatively and this agrees with Gonzalez et al [18] who showed that surgical removal of subcutaneous fat by large-volume liposuction lead to a decrease in blood glucose level over 4-weeks post-operatively and he explained this by the fact that subcutaneous abdominal fat, as a component of central adiposity, has as strong an association with insulin resistance as visceral fat, and is an important independent marker of insulin resistance in obesity [18].

Our results showed significant decrease in blood urea, serum creatinine and uric acid, many authors reported the same effects [18-20]. Concerning the serum total protein and albumin level, they showed significant decrease at 6 weeks postoperatively. This is may be attributed to redistribution and hemodilution that occurs after aspiration of large amounts of fat [21].

The lipid profile showed a significant decrease of the serum total cholesterol level 6 weeks after surgery but returned gradually to near the pre-operative levels at 4-months postoperative period and these results are nearly the same as other investigators [18,21] this is my be explained by the fact that the increased low-density lipoprotein cholesterol particles, are more susceptible to oxidation and glycosylation (particularly in the setting of diabetes) and, thus, atherogenicity [22].

Overall complication rates were low and relatively minor in nature. There were no major complications even with suctions of larger volumes of fat. These rates were also reported by nearly all authors especially among those who perform large-volume liposuction [5,11].

## Conclusion

Large-volume liposuction can be performed safely and it can produce desirable morphological and hematological changes.

## Competing interests

The authors declare that they have no competing interests.

## Authors' contributions

YS & ME & AA & AT carried out the patient selection, investigation, follow up and management.

AT & AAA-E carried out patient management, investigations, follow up, drafting of the manuscript, writing the final manuscript and provided important suggestions.

All authors read and approved the final manuscript.

## Consent

Written informed consent was obtained from all patients for publication of this manuscript and any accompanying images. A copy of the written consents is available for review by the Editor-in-Chief of this journal.
